# Renal artery and parenchymal changes after renal denervation: assessment by magnetic resonance angiography

**DOI:** 10.1007/s00330-017-4770-7

**Published:** 2017-03-07

**Authors:** Margreet F. Sanders, Pieter Jan van Doormaal, Martine M. A. Beeftink, Michiel L. Bots, Fadl Elmula M. Fadl Elmula, Jesse Habets, Frank Hammer, Pavel Hoffmann, Lotte Jacobs, Patrick B. Mark, Alexandre Persu, Jean Renkin, Giles Roditi, Wilko Spiering, Jan A. Staessen, Alison H. Taylor, Willemien L. Verloop, Eva E. Vink, Evert-Jan Vonken, Michiel Voskuil, Tim Leiner, Peter J. Blankestijn, on behalf of the European Network COordinating research on Renal Denervation (ENCOReD) Consortium

**Affiliations:** 1 Department of Nephrology and Hypertension, University Medical Center Utrecht, PO Box 85500, 3508 GA Utrecht, The Netherlands; 2 Department of Radiology, University Medical Center Utrecht, Utrecht, The Netherlands; 3 Department of Cardiology, University Medical Center Utrecht, Utrecht, The Netherlands; 4 Julius Center for Health Sciences and Primary Care, University Medical Center Utrecht, Utrecht, The Netherlands; 5 Department of Internal Medicine and Department of Cardiology, Oslo University Hospital, Ullevål Oslo, Norway; 6 Department of Radiology, Cliniques Universitaires Saint-Luc, Université Catholique de Louvain, Brussels, Belgium; 7 Section for Interventional Cardiology, Department of Cardiology, Oslo University Hospital, Ullevål Oslo, Norway; 8 Studies Coordinating Centre, Research Unit Hypertension and Cardiovascular Epidemiology, KU Leuven Department of Cardiovascular Sciences, University of Leuven, Leuven, Belgium; 9 Institute of Cardiovascular and Medical Sciences, University of Glasgow, Glasgow, Scotland UK; 10 Pole of Cardiovascular Research, Institut de Recherche Expérimentale et Clinique, Université Catholique de Louvain, Brussels, Belgium; 11 Cardiology Department, Cliniques Universitaires Saint-Luc, Université Catholique de Louvain, Brussels, Belgium; 12 Department of Radiology, Glasgow Royal Infirmary, Glasgow, UK; 13 Department of Vascular Medicine, University Medical Centre Utrecht, Utrecht, The Netherlands

**Keywords:** Renal denervation, Hypertension, Magnetic resonance angiography, Renal artery stenosis, Vascular changes

## Abstract

**Objectives:**

Relatively little is known about the incidence of long-term renal damage after renal denervation (RDN), a potential new treatment for hypertension. In this study the incidence of renal artery and parenchymal changes, assessed with contrast-enhanced magnetic resonance angiography (MRA) after RDN, is investigated.

**Methods:**

This study is an initiative of ENCOReD, a collaboration of hypertension expert centres. Patients in whom an MRA was performed before and after RDN were included. Scans were evaluated by two independent, blinded radiologists. Primary outcome was the change in renal artery morphology and parenchyma.

**Results:**

MRAs from 96 patients were analysed. Before RDN, 41 renal anomalies were observed, of which 29 mostly mild renal artery stenoses. After a median time of 366 days post RDN, MRA showed a new stenosis (25–49% lumen reduction) in two patients and progression of pre-existing lumen reduction in a single patient. No other renal changes were observed and renal function remained stable.

**Conclusions:**

We observed new or progressed renal artery stenosis in three out of 96 patients, after a median time of 12 months post RDN (3.1%). Procedural angiographies showed that ablations were applied near the observed stenosis in only one of the three patients.

***Key Points*:**

• *The incidence of vascular changes 12 months post RDN was 3.1%.*

• *No renal vascular or parenchymal changes other than stenoses were observed.*

• *Ablations were applied near the stenosis in only one of three patients.*

**Electronic supplementary material:**

The online version of this article (doi:10.1007/s00330-017-4770-7) contains supplementary material, which is available to authorized users.

## Introduction

Despite promising initial results, the efficacy of renal artery denervation (RDN) for lowering blood pressure (BP) is still subject of discussion [1, 2]. RDN, a relatively new treatment modality for hypertension, is achieved through an endovascular procedure by catheter-based radiofrequency ablation of the renal arteries [1, 3]. Because of the nature of this intervention, there is obvious concern about possible damage to the renal artery and parenchyma. This concern was fuelled by the findings of Templin et al., who analysed the incidence of renal vascular changes before and *directly* after RDN by optical coherence tomography [4]. In 24 renal arteries the authors observed a high occurrence of vascular changes; 42% of renal arteries showed vasospasm, 13% showed a dissection and there was a significant increase in the occurrence of oedema and thrombi [4]. The clinical significance of these findings is not completely understood. Several case reports demonstrated angiographically documented renal artery stenosis after RDN [5–9]. Also, clinical studies reported on vascular changes as assessed with renal artery imaging before and after RDN [10–18]. Since there is considerable variation across these studies with respect to imaging modality, follow-up time and definitions used for abnormalities, there is a clear need for more detailed information on long-term safety.

The present study aimed to investigate the safety of RDN by assessing systematically the incidence of morphological changes in renal arteries and parenchyma after RDN, compared to baseline, using magnetic resonance angiography of the renal arteries and kidneys.

## Material and methods

### Study population

The present study is an initiative of the European Network COordinating research on Renal Denervation (ENCOReD), an international collaboration of hypertension expert centres performing RDN (Table 5 of the Supplementary Material) [19]. We composed a cohort of patients who met the following criteria: age ≥18 years, treated with catheter-based radiofrequency RDN and available MRA imaging 0–12 months before RDN and MRA imaging after RDN, regardless of follow-up time. There were no further criteria with regard to indication for treatment, BP level or type of RDN catheter.

Data were collected from routine medical care or previously performed RDN studies that were approved by local medical ethics committees, in accordance with the Declaration of Helsinki and Title 45, US Code of Federal Regulations, Part 46, Protection of Human Subjects (Table 6 of the Supplementary Material) [20–25]. The methods of measurements are described in the original studies.

### RDN procedure

Assessment of indication and eligibility for RDN was performed by the treating physician or according to the centre-specific study protocol. Procedural aspects, such as location and number of ablations and which arteries to treat, were left to the operator’s discretion. RDN was performed by catheter-based radiofrequency ablation [1, 3]. Digital subtraction angiography (DSA) or normal angiography of the renal arteries was performed just before and after the procedure.

### Magnetic resonance angiography

In order to determine anatomical eligibility for RDN, contrast-enhanced MRA of the kidneys was performed at baseline. According to the centre-specific standard of care a routine follow-up MRA was performed after RDN. This MRA was offered to all consecutive patients and none of the examinations was performed based on a specific clinical indication. Details of imaging protocols and technical settings of the MRA were centre-specific and can be found in Table 7 of the Supplementary Material.

### Imaging assessment

All individual scans were anonymised and collected from the centres. Images were re-evaluated independently by two radiologists (PJD, JH) at the University Medical Centre Utrecht (UMCU), the core-lab for this study. Images were blinded for date and centre and assessed for abnormalities in the kidney or renal artery in a standardized fashion using a scoring form (provided in Table 8 of the Supplementary Material). Pre- and post-scans were evaluated in a random order and not as pairs. Abnormalities were visually identified and outcomes were scored per renal artery and per kidney. Whenever there were discrepancies between the observers, a third radiologist (TL) was consulted to reach consensus. All observed abnormalities were also evaluated by the third radiologist, unblinded for scan date. To assess whether anomalies could be attributed to the ablation, follow-up MRA examinations (for those showing abnormalities) were compared to intra-arterial DSA images, obtained during RDN.

### Outcome parameters

Primary outcome was the difference in renal artery and parenchyma morphology between baseline and follow-up. Abnormalities were defined as follows:

- Renal artery stenosis: focal luminal narrowing (category 1: stenosis of <25%, category 2: stenosis 25–49%, category 3: stenosis 50–74%, category 4: stenosis ≥75%, category 5: occlusion of the renal artery).
- Renal artery aneurysm: defined as a local increase in artery diameter of at least 20% compared to the closest adjacent normal segment.
- Renal artery dissection: presence of an intimal flap in the renal artery (flow limiting or non-flow limiting).
- Kidney infarction: sharply marginated area of hypoperfusion or cortical retraction.

Renal incidentalomas, not otherwise specified, were also scored.

### Additional assessments

We collected patient-related characteristics, both at baseline and at follow-up, and procedural details of the total population. Most of the patients were also reported in previously published studies [19, 20]. Kidney function was assessed by serum creatinine and by calculation of estimated glomerular filtration rate (Chronic Kidney Disease Epidemiology Collaboration (CKD-EPI) equation) [26].

In addition, kidney length was measured at baseline and follow-up by MR images.

### Data analysis

Data are presented as means with corresponding standard deviations (SDs), as medians with range or interquartile range (IQR), or as percentages. Primary outcome is presented as frequency of observed abnormalities and as incidence of change. A 95% confidence interval (CI) for the incidence was calculated. Paired-samples t-tests were used to assess differences in patient characteristics and kidney length before and after RDN.

Analyses were performed using the IBM SPSS Statistics for Windows, Version 21.0 (IBM Corp., Armonk, NY, USA).

## Results

### Baseline characteristics

Originating from four European RDN centres, 194 MRAs from 97 patients were considered eligible for scoring. One patient was excluded due to poor image quality, leaving 96 subjects and 192 MRAs for final analysis.

Patients were treated with RDN between November 2009 and September 2013. In all patients the indication for RDN was resistant hypertension, defined as an office systolic BP (SBP) ≥140 mmHg, despite the use of at least three BP-lowering drugs, among which was a diuretic [27]. Median time between baseline MRA and RDN was 61 days (IQR 12–114) and between RDN and follow-up MRA 366 days (IQR 213–397). In total, the median time between the two MRAs was 434 days (IQR 358–502). Table 1 shows the patient and procedural characteristics. Ninety-two patients were treated with the Symplicity™ RDN catheter (Medtronic Inc., Santa Rosa, CA, USA). Other devices used are EnligHTN™ multi-electrode RDN system (St Jude Medical, St Paul, MN, USA) (twice), OneShot™ RDN System (Covidien, Mansfield, MA, USA) and Vessix™ RDN System (Boston Scientific, Marlborough, MA, USA). The mean number of ablations per artery was 6 ± 1.4. No major peri-procedural adverse events occurred. Mean office BP at baseline was 187/104 ± 30/16 mmHg and mean baseline eGFR was 81 ± 19 ml/min/1.73 m^2^.

**Table 1:** Baseline characteristics of the study population

	All patients (*n* = 96)
Age (years)	57 (range 35–80)
Sex, % male/female	55/45
Caucasian, %	93
Body mass index (kg/m^2^)	29 ± 5
Co-morbidity, %	
Diabetes mellitus type 2	24
Cardiovascular diseases	19
Cerebrovascular diseases	7
Prescribed antihypertensive drugs, no.	4 (IQR 3–6)
Office SBP/DBP (mmHg)	187/104 ± 30/16
Office heart rate (bpm)	73 ± 14
eGFR (ml/min/1.73 m^2^)	81 ± 19
Patients with accessory renal arteries, no.﻿ (%)	28 (29)
Categorization of renal artery anatomy	
Eligible anatomy, % (A1 or A2)	85
Non-eligible, % (A3, B1 or B2)	15
Renal arteries treated, no.	197
Time baseline MRA –RDN in days	61 (IQR 12–114)
Symplicity device used, no.	92
Other device used, no.	4
Ablations per artery, no.	6.0 ± 1.4
Major procedural complications, %	0

Data are presented as mean ± SD, unless stated otherwise

*SBP* systolic blood pressure; *DBP* diastolic blood pressure; *bpm* beats per minute; *eGFR* estimated glomerular filtration rate; *MRA* magnetic resonance angiography; *RDN* renal denervation

### Renal artery anatomy

In total, 192 kidneys and 229 renal arteries were evaluated. Twenty-eight out of 96 patients had accessory renal arteries (Table 1). Except for one patient, all patients were treated in both main renal arteries. Sixteen percent of the accessory renal arteries were treated with RDN. Based on the OKADA classification, a tool to assess anatomical eligibility for RDN, 15% of the patients had one or two kidneys that would be considered ineligible for the procedure [28].

### Renal artery and parenchymal findings

Table 2 shows the scoring results of the MRAs pre and post RDN separately. All individual abnormalities were counted. Before RDN, 31 vascular abnormalities in 25 patients were observed, which represents a prevalence of 26.0% (95% CI 17.1–35.0) of the patients (25 out of 96) and 15.7% (95% CI 10.7–20.8) of the treated renal arteries (31 out of 197). Twenty-three patients had a renal artery stenosis at baseline; 13 patients had a lumen reduction of <25%, nine patients 25–49% and one patient >50% (in an untreated accessory renal artery). Six patients showed a second renal artery stenosis, of which two had a lumen reduction of 25–49% and the remainder <25%. The remaining lumen diameter was considered sufficient for RDN by the treating physician. Two of the 25 patients with pre-existing renal vascular abnormalities had an aneurysm.

**Table 2:** Renal vascular (only treated arteries) and parenchymal observations, after assessment by the senior radiologist

	Pre	Post
Renal artery stenosis	29	31
Lumen reduction <25%	17	15
25–49%	11	14
50–74%	1	2
>74%	0	0
Of which second stenosis	6	6
Renal artery aneurysm	2	2
Renal artery dissection	0	0
Kidney infarction	2	2
<20%	1	1
>20%	1	1
Kidney-related incidentalomas	8	8
Cortical tissue loss	5	5
Other focal lesion	2	2
Unilateral small kidney	1	1

Absolute numbers are presented

After RDN, in two patients a new renal artery stenosis was observed (lumen reduction in both cases 25–49%) and in one patient there was progression of a pre-existing lumen reduction (from <25% to 50–74%). Two of the three patients with vascular changes were treated with the Symplicity™ catheter and one with the EnligHTN™ multi-electrode system.

In both patients with an aneurysm at baseline, the renal artery diameter did not increase further during follow-up. Throughout the cohort, no new aneurysms or renal artery dissections were observed. In two patients, kidney tissue infarction was observed at baseline and, with the same severity, after RDN.

In total, the incidence of vascular changes over the observed time period after RDN was 3.1% (95% CI −0.4 to 6.7) of treated patients and 1.5% (95% CI −0.2 to 3.2) of treated arteries.

Mean kidney length at baseline was 113 ± 12 mm on the left side and 109 ± 12 mm on the right side (Table 9 of the Supplementary Material). Kidney length did not significantly change after RDN (*p* = 0.78 on the left and *p* = 0.68 on the right).

**Fig. 1:**
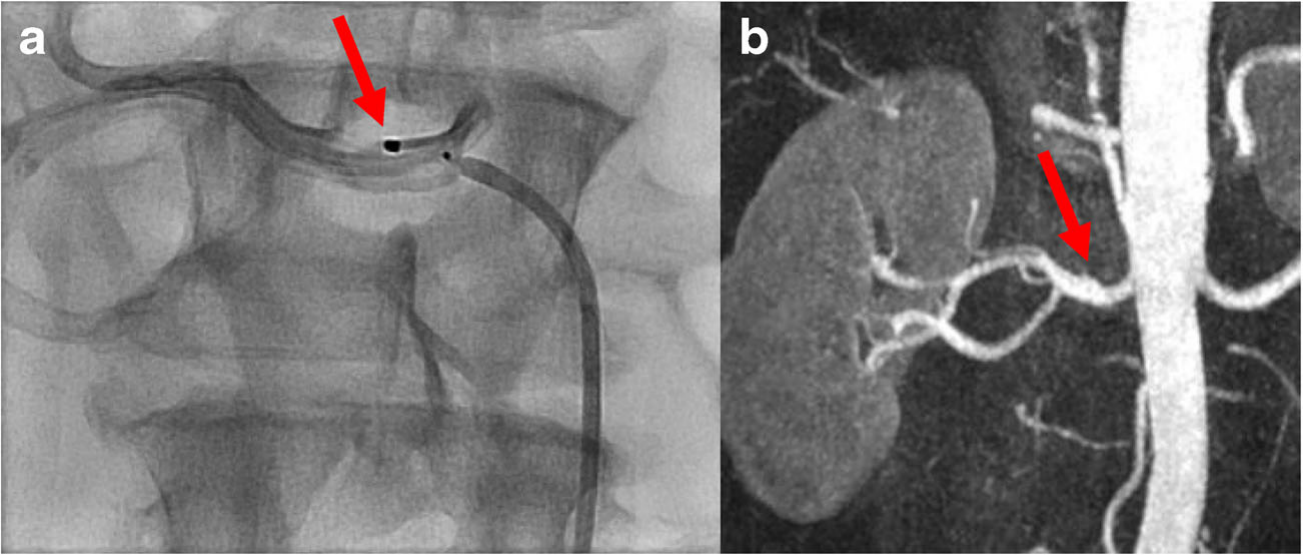
Radiofrequency ablation point on the procedural angiography (red arrow, panel **a**) near the location where on the magnetic resonance angiography (MRA) post renal denervation (RDN) a new renal artery stenosis was observed (red arrow, panel **b**)

To assess the possible relationship between vascular changes and the radiofrequency ablations, we analysed the DSA images performed during RDN. In only one of the three patients with vascular changes after RDN we could conclude that ablations were applied near the location where on the follow-up MRA a new stenosis was observed (Fig. 1). Furthermore, we concluded that findings on the pre-procedural MRA corresponded to the DSA images.

### Follow-up parameters

Table 3 shows patient-related parameters during the follow-up MRA. Mean office SBP and DBP were significantly lower compared to baseline. There were no differences in prescribed medication. Importantly, kidney function remained unchanged.

**Table 3:** Patient-related parameters during the follow-up MRA

	All patients (*n* = 96)
Body mass index (kg/m^2^)	29 ± 5
No. of antihypertensive drugs	4 (IQR 3–5)
Office SBP/DBP (mmHg)	164/96 ± 25/16*
Office heart rate (bpm)	70 ± 12
eGFR (ml/min/1.73 m^2^)	82 ± 19
Time RDN – follow-up MRA in days	366 (IQR 213–397)

Data are presented as mean ± standard deviation or as median with interquartile range (IQR)

*SBP* systolic blood pressure, *DBP* diastolic blood pressure, *bpm* beats per minute, *eGFR* estimated glomerular filtration rate, *MRA* magnetic resonance angiography, *RDN* renal denervation

*Significant reduction, compared to baseline (*p* < 0.001)

Table 4 presents the individual characteristics of the patients with newly observed or progressed renal artery stenosis. Only one of the three patients showed a marked decrease in BP. No other clinical signs (i.e. impaired kidney function) of renal artery stenosis were reported. Also in these patients, kidney length did not change after RDN.

**Table 4:** Characteristics of patients with vascular changes

	Patient 1	Patient 2	Patient 3
Office SBP/DBP (mmHg)			
Baseline	197/115	180/95	173/111
Follow-up	183/99	190/90	186/121
eGFR (mL/min/1.73 m^2^)			
Baseline	>90	48	75
Follow-up	>90	66	>90
Time MRA1 – RDN (days)	90	177	207
Time RDN – MRA2 (days)	357	355	192
Ablations in artery of interest	8	4	5
Lumen reduction, %			
Baseline	N/A	N/A	<25%
Follow-up	25–49%	25–49%	50–74%
Artery type	Main	Main	Main
Arterial segment	2	1	1
Kidney length, side stenosis (mm)			
Baseline	125	105	94
Follow-up	123	103	98

Data are presented as absolute numbers or percentage

*SBP* systolic blood pressure, *DBP* diastolic blood pressure, *eGFR* estimated glomerular filtration rate, *MRA* magnetic resonance angiography, *RDN* renal denervation, *N/A* not applicable

## Discussion

This study reports on the largest population in which renal changes after RDN were systematically assessed by MRA. The incidence of new or progression of pre-existing vascular abnormalities was 3.1% (95% CI −0.4 to 6.7) of treated patients and 1.5% (95% CI −0.2 to 3.2) of treated arteries after a median follow-up of 12 months after RDN. Two patients had a new renal artery stenosis and one patient showed progression of pre-existing stenosis. No new or progressed aneurysms, dissections or renal parenchymal anomalies were observed.

The incidence of renal changes after RDN is described in many studies and mostly varies from 0% to 4.4%, with a few outliers of 24% and even 30.7% [8, 10–18]. The ability to compare the incidences reported in the literature is hampered by the large variations in study design, such as imaging modality used, ablation catheter used and the morphological abnormalities that were of interest to the investigators. Few studies made a special effort to primarily investigate the renal artery changes after RDN [14, 16, 17].

Lambert et al. showed an incidence of 2.6% (95% CI −1.0 to 6.2) (two out of 76 patients) of new or progressed renal artery stenosis, using MRA or CTA, 6 months after RDN [16]. In none of these cases lumen reduction exceded 70%. In the mentioned study, renal artery imaging was also systematically evaluated as part of the primary study aim. It is unclear whether they observed other renal abnormalities. As in our population, the Symplicity catheter was used most frequently with a comparable mean number of ablations per artery.

Zhang et al. also specifically investigated the effects of RDN on the renal arteries, using a multidetector spiral CTA [14]. In a population of 39 treated patients, no renal artery stenoses, aneurysms or dissections were observed before and 12 months after RDN (incidence 0% [95% CI −4.4 to 4.4]). Interestingly, they showed an increase in cases with renal atherosclerosis and a significant increase in plaque burden in the 38 control patients, while there were no differences in the RDN group. In the Prague-15 study, an RCT comparing the BP-lowering efficacy of RDN to pharmacotherapy, also only (minimal) progression of atherosclerotic lesions was observed, in 24% of the 37 treated patients by CTA assessment after 12 months [10].

Recently, Schmid et al. reported on a cohort of 51 resistant hypertensive patients, treated with RDN [17]. In accordance with our study, an MRA was performed before RDN and after a median follow-up of 12 months, and a median number of six ablations was applied. The investigators did not observe any anomalies in renal arteries or parenchyma.

Contemporary MRA technique is known for its high sensitivity and specificity (at least 90% and 92%, respectively) for the detection of main renal artery stenosis, comparable to CTA [29–31]. In RDN studies, renal vascular changes were often assessed using duplex ultrasonography [11, 12, 15, 18]. However, studies that primarily aimed at investigating changes in renal arteries using MRA or CTA observed incidences that did not differ much from those found using duplex ultrasonography [11, 12, 14–18]. An explanation for this might be a lack of precision, due to small sample sizes and a small number of events.

Based on the evaluation of the procedural angiographies, we concluded that only in one patient the newly observed stenosis was located in an ablated area. A relationship with the procedure could therefore not be excluded. In the other two patients, there was no reason to believe that the new or progressed lumen reduction after RDN may have been a result of the ablations (very proximal stenosis and more distally ablated). The natural history of renal vascular anomalies within 1 year in hypertensive patients who had no stenosis at baseline is not often investigated. In 1998, Caps and co-workers reported a cumulative incidence of progression to ≥60% stenosis of at least 5% after 1 year in patients who were initially wrongly suspected of having atherosclerotic renal artery stenosis (investigated by duplex ultrasonography) [32]. The cumulative incidence of progression in patients with pre-existing stenosis was almost five times higher. Two other studies showed that in a population of resistant hypertensive patients with relatively high vascular morbidity, occurrence or progression of renal artery atherosclerosis is very likely [33, 34]. For a correct interpretation of the currently presented results, a comparison with (randomized) control patients and differentiation between atherosclerosis and other causes of renal artery stenosis would be useful. However, this differentiation is radiologically challenging.

It is important to realize that most RDN procedures were performed with the Symplicity catheter and that the mean number of ablations was six per artery. Recent studies have shown that with this device a variable degree of denervation is obtained [35, 36]. This may have influenced our findings since one may assume that insufficient ablation energy could result in less renal artery or kidney injury in some of these patients. Due to the small number of abnormalities in our study, we were not able to investigate the relationship between number of ablations and the occurrence of abnormalities. Presently, newer devices are available, a higher number of ablation points is advised and different technologies for denervation have been introduced. The incidence of vascular changes following treatment with these novel devices should therefore be a subject of interest in future studies. The imaging protocols presented in this paper may serve as a basis for these studies.

The strengths of this study are the multicentre design, the assessment by MRA and the standardized blinded evaluation of scans. We made a great effort to objectively review the MRAs, which improved the quality of our results. Importantly, all scans were performed according to the centre-specific standard of care, which means that none of the MRAs had a clinical indication. This results in a good reflection of the real incidence of vascular changes after RDN for this device and dosage. Also, the multicentre aspect and consequently differences in scan parameters contribute to better representative results.

An important limitation of this study is the absence of a reference group, i.e. a group of individuals suitable for RDN, yet not receiving RDN. As discussed above, vascular changes could be due to radiofrequency ablation or just be a natural history of disease. Although MRA has sufficient sensitivity to detect stenoses, this technique does not allow for differentiation between atherosclerosis and other causes of renal artery stenosis. Finally, we cannot rule out that selection based on complete follow-up (availability of two MRAs) may have influenced our results, although follow-up MRAs had no clinical indication. An overestimation of renal changes after RDN could theoretically be the consequence.

In conclusion, based on the largest population in which renal changes after RDN were systematically assessed by MRA, the total incidence of vascular changes after a median time of 12 months post-renal denervation was 3.1% (95% CI −0.4 to 6.7). The procedural angiographies showed that ablations were applied near the observed stenosis in only one of the three patients. The results of this study indicate that the risks of RDN to the renal arteries and parenchyma appear to be limited.

## Electronic supplementary material

Below is the link to the electronic supplementary material.

ESM 1 (DOCX 22 kb)

## Abbreviations and acronyms

BP: Blood pressure
CKD-EPI: Chronic Kidney Disease Epidemiology Collaboration
DBP: Diastolic blood pressure
ENCOReD: European Network COordinating research on Renal Denervation
RDN: Renal denervation
SBP: Systolic blood pressure

## References

[1] Krum H, Schlaich M, Whitbourn R (2009). Catheter-based renal sympathetic denervation for resistant hypertension: a multicentre safety and proof-of-principle cohort study. Lancet.

[2] Esler MD, Krum H, Sobotka PA, Schlaich MP, Schmieder RE, Bohm M (2010). Renal sympathetic denervation in patients with treatment-resistant hypertension (The Symplicity HTN-2 Trial): a randomised controlled trial. Lancet.

[3] Blessing E, Esler MD, Francis DP, Schmieder RE (2013). Cardiac ablation and renal denervation systems have distinct purposes and different technical requirements. JACC Cardiovasc Interv.

[4] Templin C, Jaguszewski M, Ghadri JR (2013). Vascular lesions induced by renal nerve ablation as assessed by optical coherence tomography: pre- and post-procedural comparison with the Simplicity catheter system and the EnligHTN multi-electrode renal denervation catheter. Eur Heart J.

[5] Lambert T, Blessberger H, Grund M, Steinwender C (2014) Late renal artery stenosis after percutaneous renal denervation. J Cardiovasc Med (Hagerstown) 15 10.2459/JCM.0000000000000095 25090272

[6] Vonend O, Antoch G, Rump LC, Blondin D (2012). Secondary rise in blood pressure after renal denervation. Lancet.

[7] Kaltenbach B, Id D, Franke JC (2012). Renal artery stenosis after renal sympathetic denervation. J Am Coll Cardiol.

[8] Persu A, Sapoval M, Azizi M (2014). Renal artery stenosis following renal denervation: a matter of concern. J Hypertens.

[9] Bhamra-Ariza P, Rao S, Muller DW (2014). Renal artery stenosis following renal percutaneous denervation. Catheter Cardiovasc Interv.

[10] Rosa J, Widimsky P, Waldauf P (2016). Role of adding spironolactone and renal denervation in true resistant hypertension: one-year outcomes of randomized PRAGUE-15 study. Hypertension.

[11] Krum H, Schlaich MP, Bohm M (2013). Percutaneous renal denervation in patients with treatment-resistant hypertension: final 3-year report of the Symplicity HTN-1 study. Lancet.

[12] Bhatt DL, Kandzari DE, O'Neill WW (2014). A controlled trial of renal denervation for resistant hypertension. N Engl J Med.

[13] Papademetriou V, Tsioufis CP, Sinhal A (2014). Catheter-based renal denervation for resistant hypertension: 12-month results of the EnligHTN I first-in-human study using a multielectrode ablation system. Hypertension.

[14] Zhang ZH, Yang K, Jiang FL, Zeng LX, Jiang WH, Wang XY (2014). The effects of catheter-based radiofrequency renal denervation on renal function and renal artery structure in patients with resistant hypertension. J Clin Hypertens (Greenwich).

[15] Mahfoud F, Cremers B, Janker J (2012). Renal hemodynamics and renal function after catheter-based renal sympathetic denervation in patients with resistant hypertension. Hypertension.

[16] Lambert T, Nahler A, Reiter C (2015). Frequency of renal artery stenosis after renal denervation in patients with resistant arterial hypertension. Am J Cardiol.

[17] Schmid A, Schmieder R, Lell M (2016). Mid-term vascular safety of renal denervation assessed by follow-up MR imaging. Cardiovasc Intervent Radiol.

[18] Esler MD, Bohm M, Sievert H (2014). Catheter-based renal denervation for treatment of patients with treatment-resistant hypertension: 36 month results from the SYMPLICITY HTN-2 randomized clinical trial. Eur Heart J.

[19] Persu A, Jin Y, Baelen M (2014). Eligibility for renal denervation: experience at 11 European expert centers. Hypertension.

[20] Persu A, Jin Y, Azizi M (2014). Blood pressure changes after renal denervation at 10 European expert centers. J Hum Hypertens.

[21] Vink EE, de Boer A, Verloop WL (2015). The effect of renal denervation on kidney oxygenation as determined by BOLD MRI in patients with hypertension. Eur Radiol.

[22] Sanders MF, Blankestijn PJ, Voskuil M (2016). Safety and long-term effects of renal denervation: rationale and design of the Dutch registry. Neth J Med.

[23] Persu A, Azizi M, Jin Y (2014). Hyperresponders vs. nonresponder patients after renal denervation: do they differ?. J Hypertens.

[24] Fadl Elmula FE, Hoffmann P, Larstorp AC (2014). Adjusted drug treatment is superior to renal sympathetic denervation in patients with true treatment-resistant hypertension. Hypertension.

[25] Fadl Elmula FE, Hoffmann P, Fossum E (2013). Renal sympathetic denervation in patients with treatment-resistant hypertension after witnessed intake of medication before qualifying ambulatory blood pressure. Hypertension.

[26] Levey AS, Stevens LA, Schmid CH (2009). A new equation to estimate glomerular filtration rate. Ann Intern Med.

[27] Mancia G, Fagard R, Narkiewicz K (2013). 2013 ESH/ESC Guidelines for the management of arterial hypertension: the Task Force for the management of arterial hypertension of the European Society of Hypertension (ESH) and of the European Society of Cardiology (ESC). J Hypertens.

[28] Okada T, Pellerin O, Savard S (2015). Eligibility for renal denervation: anatomical classification and results in essential resistant hypertension. Cardiovasc Intervent Radiol.

[29] Slanina M, Zizka J, Klzo L, Lojik M (2010). Contrast-enhanced MR angiography utilizing parallel acquisition techniques in renal artery stenosis detection. Eur J Radiol.

[30] Rountas C, Vlychou M, Vassiou K (2007). Imaging modalities for renal artery stenosis in suspected renovascular hypertension: prospective intraindividual comparison of color Doppler US, CT angiography, GD-enhanced MR angiography, and digital substraction angiography. Ren Fail.

[31] Leiner T, de Haan MW, Nelemans PJ, van Engelshoven JM, Vasbinder GB (2015). Contemporary imaging techniques for the diagnosis of renal artery stenosis. Eur Radiol.

[32] Caps MT, Perissinotto C, Zierler RE (1998). Prospective study of atherosclerotic disease progression in the renal artery. Circulation.

[33] Daugherty SL, Powers JD, Magid DJ (2012). Incidence and prognosis of resistant hypertension in hypertensive patients. Circulation.

[34] Olin JW, Melia M, Young JR, Graor RA, Risius B (1990). Prevalence of atherosclerotic renal artery stenosis in patients with atherosclerosis elsewhere. Am J Med.

[35] Vink EE, Goldschmeding R, Vink A, Weggemans C, Bleijs RL, Blankestijn PJ (2014). Limited destruction of renal nerves after catheter-based renal denervation: results of a human case study. Nephrol Dial Transplant.

[36] Tzafriri AR, Keating JH, Markham PM (2015). Arterial microanatomy determines the success of energy-based renal denervation in controlling hypertension. Sci Transl Med.

