# Supplementary material for: Renal artery and parenchymal changes after renal denervation: assessment by magnetic resonance angiography
Source: Eur Radiol. 2017 Mar 7;27(9):3934–41. doi: 10.1007/s00330-017-4770-7 (PMC5544801; doi:10.1007/s00330-017-4770-7)
Supplement: Supplementary file 1 — (DOCX 22 kb) [file 330_2017_4770_MOESM1_ESM.docx]

Electronic Supplementary Material

Table 5. European Network COordinating research on Renal Denervation (ENCOReD)

Brussels, Belgium – Alexandre Persu, Frank Hammer, Sandrine Horman, Joëlle Kefer, Jean-Philippe Lengelé, Jean-Benoit Le Polain de Waroux, Agnès Pasquet, Jean Renkin, Christophe Scavée, Francesca Severino, Christophe Beauloye, Jean-Louis Vanoverschelde.

Glasgow, Scotland, UK – Patrick B Mark, Christian Delles, Anna F Dominczak, Rhian Touyz, Sandosh Padmanabhan, Marie Freel, Alan Jardine, Ram Kasthuri, Jon Moss, Giles Roditi, Adrian Brady.

Leuven, Belgium – Jan A Staessen, Yu-Mei Gu, Azusa Hara, Lotte Jacobs, Yu Jin, Tatiana Kuz-netsova, Lutgarde Thijs, Fang-Fei Wei, Wen-Yi Yang, Zhen-Yu Zhang.

Oslo, Norway – Fadl Elmula M Fadl Elmula, Eigil Fossum, Ulla Hjørnholm, Pavel Hoffmann, Aud Høieggen, Vibeke Kjær, Sverre E Kjeldsen, Anne CK Larstorp, Ingrid Os, Morten Rostrup, Aud Stenehjem.

Paris, France – Michel Azizi, Marc Sapoval

Utrecht, The Netherlands – Peter J Blankestijn, Martine MA Beeftink, Michiel L Bots, Rosa L de Jager, Wilko Spiering, Evert-Jan Vonken, Michiel Voskuil, Margreet F Sanders

| Table 6. Origin of patients | | |
| --- | --- | --- |
| Participating center | N | Pubmed ID |
| University Medical Center Utrecht, Utrecht, The Netherlands | 64 | 25479032  26819356 |
| Cliniques Universitaires Saint-Luc, Brussels, Belgium | 14 | 24067345 |
| University of Glasgow, Glasgow, Scotland, UK | 12 | 25375391 24067345 |
| Oslo University Hospital, Ullevål, Oslo, Norway | 7 | 23836798 24591332 |

| Table 7. MRA parameters and MRA sequence parameters | | | | | | | | |  |
| --- | --- | --- | --- | --- | --- | --- | --- | --- | --- |
|  | Utrecht | | Brussels | | Glasgow | | Oslo | |  |
| MR brand and model | | Ingenia and Achieva Multitransmit, Philips Healthcare | | GE Healthcare 450w GEM, DV25 | | Siemens Magnetom Verio, Siemens Medical Solutions | | Intera Gyroscan NT and  Achieva (Nova Dual), Philips Healthcare | |
| Software release | 3.2.3.2 (3.0T),  5.1.7.2 (1.5T) | | Version DV25  Optima MR450W | | VB17 | | 14.1.4.6 and 3.2.3.2. | |  |
| Field strength | 1.5T and 3.0T | | 1.5T | | 3.0T | | 1.5T | |  |
| Gradient strength (mT/m) | 40 (3.0T), 33 (1.5T) | | 44 | | 45 | | 23 and 33 | |  |
| Slew rate (mT/m/s) | 200 (3.0T), 200 (1.5T) | | 200 | | 200 | | 105 and 180 | |  |
| Contrast agent name | Gadovist | | Gadovist | | Gadovist | | MultiHance | |  |
| Contrast dosage | 0.15mmol/kg + 3mmol | | 0.2mmol/kg | | 0.2mmol/kg | | 20 ml (15 ml < 60 kg) | |  |
| Injection rate (mL/s) | 2 | | 1.2 | | 2 | | 2 | |  |
| TE (ms) | 1.5 | | 1.8 | | 1.2 | | 1.62 and 1.29 | |  |
| TR (ms) | 4.6 | | 5.2 | | 3.2 | | 4.5 and 3.9 | |  |
| flip angle (degrees) | 35 | | 30 | | 25 | | 35 | |  |
| receiver band width (Hz) | 1273/pixel (3.0T), 437/pixel (1.5T) | | 50,000 | | 660/pixel | | 361.9/pixel and 360.1/pixel | |  |
| Field of view (FH x LR; mm) | 290x228 | | 400x320 | | 360x247 | | 450x346 | |  |
| Matrix (Frequency x Phase) | 356x281 | | 384x256 | | 448x277 | | 333x416 and 312x327 | |  |
| Acquired slice thickness (mm) | 2.4 | | 2.4 | | 0.90 | | 2.6 and 2.0 | |  |
| Acquired voxel size (mm3) | 0.81x0.81x2.4 | | 2.4x0.96x0.64 | | 0.89x0.80x0.90 | | 1.08x1.08 and 1.05x1.06 | |  |
| Reconstructed voxel size (mm3) | 0.57x0.56x1.2 | | 1.2x0.96x0.64 | | 0.70x0.70x0.45 | | 0.80x0.81 and 0.67x0.68 | |  |
| k-Space filling(centric or linear) | centric | | Linear | | centric | | centric | |  |
| Acquisition time (sec. breath-hold) | 17.9 | | 19 | | 20 | | 22 and 21 | |  |

Table 8. Scoring items

- Number of renal arteries
- Categorization of the renal arteries (OKADA)
- Image quality: possible or not possible to score
- Kidney infarction: yes / no
- Kidney- related incidentalomas: yes / no. If yes: specify
- Per segment, per artery, per kidney:
   Renal artery stenosis: <25, 25-49%, 50-74%, >74%, occlusion.
   Renal artery aneurysm: yes / no
   Renal artery dissection: yes / no
   In case of the presence of a dissection: flow limiting / non-flow limiting
- Segment definitions:
   Segment 1: ostium, < 1 cm from the aorta
   Segment 2: ≥1 to < 3 cm from the aorta
   Segment 3: ≥ 3 cm from the aorta till bifurcation of the renal artery
- Kidney length in mm

| Table 9. Kidney length | | | | |
| --- | --- | --- | --- | --- |
|  | N | Pre | | Post |
| Kidney length | | | | |
| Left kidney (mm) | 96 | | 112.7 ±12.0 | 112.6 ±11.8 |
| Right kidney (mm) | 96 | | 109.4 ±11.7 | 109.7 ±11.6 |

*Numbers are presented as mean ±SD. Kidney length did not significantly change after RDN.*
